# New Obstructive Sleep Apnoea Multi‐Night Diagnostic Devices. Is a Single Night of Measurement Now Considered Enough?

**DOI:** 10.1002/resp.70263

**Published:** 2026-05-24

**Authors:** Thomas J. Altree, Robert J. Adams

**Affiliations:** ^1^ Flinders Health and Medical Research Institute – Sleep Health, Flinders University Adelaide South Australia Australia; ^2^ Respiratory and Sleep Unit, The Queen Elizabeth Hospital, Central Adelaide Local Health Network Adelaide South Australia Australia; ^3^ Respiratory and Sleep Service, Southern Adelaide Local Health Service Adelaide South Australia Australia

**Keywords:** apnoea‐hypopnea index (AHI), night‐to‐night variability, obstructive sleep apnoea (OSA), polysomnography

## Abstract

– The apnoea‐hypopnoea index (AHI) varies from night to night.
– Multi‐night testing improves diagnostic accuracy.
– Emerging multi‐night diagnostic devices offer practical advantages but require further validation.
– A single night AHI should be interpreted as an estimate rather than a definitive value, alongside clinical judgement.

Sleep is a dynamic process that varies from one night to another. Even the soundest of sleepers experience substantial changes in sleep duration from night to night [1]. The severity of obstructive sleep apnoea (OSA), a common sleep disorder characterised by repetitive narrowing of the upper airway, also varies from night to night [2]. OSA severity is measured by the apnoea‐hypopnoea index (AHI), a count per hour of the number of reductions in airflow lasting ≥ 10 s associated with either an oxygen desaturation or an awakening from sleep during polysomnography (PSG). OSA is categorised as either mild (AHI of 5 to < 15 events/h), moderate (15 to < 30 events/h), or severe (≥ 30 events/h). In current clinical practice, OSA treatment decisions rely heavily on the AHI.

The AHI has traditionally been measured by in‐laboratory PSG, which is considered the ‘gold‐standard’ test for OSA due to the comprehensive nature of data recorded. It has also traditionally been used as the comparator for other diagnostic sleep methods, such as home‐based sleep apnoea tests. It remains the recommended test when there is clinical suspicion for complex sleep disorders, or in people with significant comorbidities. However, in‐laboratory PSG is costly, labour‐intensive, and many people have limited access to a sleep laboratory service. Home sleep apnoea tests vary considerably in the data they provide, but when the pre‐test probability for OSA is high, guidelines recommend home sleep apnoea testing with a technically adequate device as a reasonable alternative to in‐lab PSG [3]. In most cases, the AHI from a PSG or home sleep apnoea test is measured on a single night.

A fundamental problem with this approach is that the AHI varies from night to night. In people with known or suspected OSA who have more than one diagnostic sleep study, the AHI varies by over 10 events/h in 41% (95% Confidence Interval (CI) [27%–57%]) of people, and the OSA severity category changes in almost half of all people tested (49%, 95% CI [32% to 65%]) [2].

There are several causes of variability that may affect the single night AHI. The causes are primarily biological, rather than related to variability in the accuracy of the sleep test. Sleep position and sleep stages vary night to night. OSA is worse in the supine position and during REM sleep [4]. Drugs and alcohol also have an impact. Even a moderate amount of alcohol prior to sleep significantly increases the AHI in known snorers [5]. Relevant anatomical factors such as upper airway collapsibility and nasal congestion can also fluctuate on a nightly basis [6]. Although the PSG captures a rich array of data during a single night of sleep, reliance on the AHI risks misclassification of OSA severity due to night‐to‐night variability (N2NV). Such variability is potentially addressed by new multi‐night sleep apnoea diagnostic devices for use in the home.

Sleep apnoea testing devices suitable for multi‐night assessment can be broadly categorised into wearable and contactless (“nearable”) systems. Wearable devices include level III home sleep apnoea tests and peripheral arterial tonometry‐based systems, which typically measure combinations of airflow, respiratory effort, oxygen saturation, heart rate and autonomic signals, enabling direct or semi‐direct estimation of the AHI. Electroencephalography is typically not measured. These devices have demonstrated moderate‐to‐high sensitivity and specificity for detecting moderate‐to‐severe OSA in validation studies against polysomnography [7, 8]. In contrast, nearable devices are contactless systems (e.g., under‐mattress sensors) that estimate sleep and breathing patterns using signals such as movement, including micro‐movements produced by the cardiovascular system's mechanical forces, respiratory motion, and in some cases, breath sounds. These devices generally do not measure oxygen saturation directly, but rather use complex algorithms to infer respiratory events. Similar to wearable devices, there is a moderate‐to‐high single‐night sensitivity and specificity for moderate to severe OSA compared with in‐laboratory polysomnography [9]. Additional wearable technologies, such as oximetry‐based rings, also enable multi‐night testing but typically estimate AHI using simplified signals combined with machine‐learning approaches [10].

Under‐mattress (nearable) sleep monitoring device data demonstrate that the likelihood of a single night AHI being incorrect is highest in mild and moderate OSA. In an analysis of rates and severity of OSA in over 65,000 adults using an under‐mattress nearable, a single night AHI result characterised mild OSA correctly in only 54% of cases, and moderate OSA in only 52% [11]. These are important findings, because OSA treatment decisions and many positive airway pressure device reimbursement schemes are highly influenced by whether the AHI is higher or lower than 15 events per hour [12, 13]. Reliance on a single night AHI risks selection of potentially inappropriate treatment, especially when the AHI is near the border of AHI severity categories.

Due to variability in the AHI, single night testing also risks misdiagnosis at the individual level and underestimates prevalence at the population level. Single night tests miss approximately 10%–20% of OSA cases [2, 11, 14]. Diagnosis rates improve with multiple nights of testing [2, 11, 14]. Considering that single night tests can give false negative results or misclassify OSA severity, multi‐night testing is a worthwhile strategy in cases where the results of a single night test do not align with the clinical symptoms.

If multiple nights of data reduce the risk of misclassification, then how many nights should be tested? It is clearly not practical to perform in‐laboratory sleep studies night after night. Home‐based sleep apnoea testing offers the flexibility to test multiple nights in a row. Under‐mattress nearable device OSA diagnostic accuracy improved from 1 night to 14 nights of data. Beyond 14 nights, there was no significant improvement in false negative or false positive rates [11]. Three nights of testing improved OSA classification accuracy versus one night with both a level III home sleep apnoea test [14] and a peripheral arterial tonometry device [15]. However, at present, there are insufficient data to make definitive statements on the optimal number of nights to test.

It is also important to consider the accuracy and reproducibility of multi‐night testing devices, especially when the AHI is inferred using algorithms, as in the case of some wearables such as novel oximeter rings and under mattress devices, rather than directly measured airflow and respiratory effort. Of the various devices available there is currently no one clear stand‐out. There is limited evidence assessing the clinical outcomes associated with their use. Studies comparing home‐based devices to in‐lab polysomnography have generally been small and underrepresent certain groups including the elderly and people with comorbidities that influence breathing during sleep. Large Australian clinical trials addressing some of these limitations are underway (e.g., ACTRN12625000279426 and ACTRN12625000032459) [16], and it is likely that technological advances will improve the accuracy of these devices over time, but until there are rigorous data, including in underrepresented groups, clinicians must keep in mind that the results of these devices may not always accurately reflect the AHI. As ever, clinicians must consider the overall clinical picture, not just the AHI result.

A potential additional benefit of non‐invasive nightly testing is the ability to track changes in OSA severity over time. Such data could show changes in AHI with weight increase or decrease, or an abrupt improvement with the addition of OSA therapy where adherence and efficacy data aren't easily available, such as mandibular advancement splints. An example is shown in Figure 1, where an individual's AHI is tracked over time with an under‐mattress device as well as morning blood pressure measurements. The initial high AHI with high N2NV as well as elevated blood pressure are reduced with therapy. Over time with variable therapy use, N2NV in the AHI returns along with some increase in blood pressure. Additionally, the degree of night‐to‐night AHI variability may in itself be a clinically important phenotype that cannot be identified by current standard diagnostic pathways. High AHI night‐to‐night variability is associated with uncontrolled hypertension and potentially warrants more aggressive OSA treatment [17].

**FIGURE 1:**
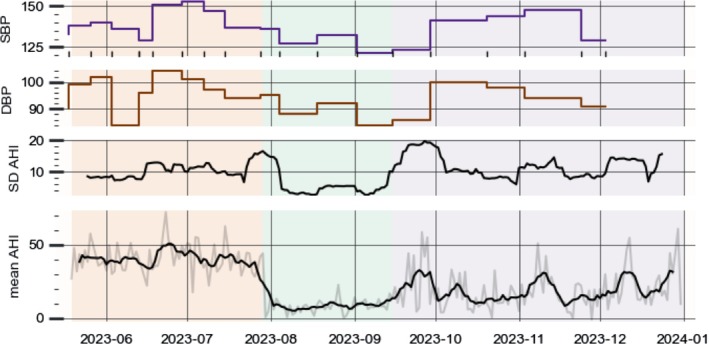
Night‐to‐night variability (N2NV) in AHI over time on and off therapy, measured with a Withings Sleep Analyser. Panel 1 (2023–06 to 2023–08) shows high AHI and standard deviation of AHI (i.e., N2NV) with elevated blood pressure. Panel 2 (2023–08 to 2023–10) shows reduced AHI, N2NV and blood pressure with therapy. Panel 3 (2023–10 to 2024–01) shows variable AHI, N2NV and elevated blood pressure, with varying therapy usage.

There is clearly value in measuring the AHI on multiple nights. At present, there are limitations in the accuracy of multi‐night diagnostic devices, but the technology is expected to improve. Multi‐night testing does not replace traditional in‐laboratory PSG in complex cases and may not be necessary in cases with high pre‐test probability for severe OSA, but it does appear to be particularly valuable in those with mild to moderate OSA. No matter what test is used, clinical judgement remains central to decisions regarding treatment in OSA. Due to the presence of night‐to‐night variability in OSA severity, the AHI should be considered as a rough indicator of OSA severity, rather than an absolute, definitive value.

## Funding

The authors have nothing to report.

## Conflicts of Interest

R.J.A. has received research grants from the NHMRC, MRFF, Lifetime Support Authority, The Hospital Research Foundation, and ResMed Foundation, and equipment from ResMed, Philips, Withings and ApniMed. T.J.A. has nothing to disclose.
